# Behavior of Smooth Muscle Cells under Hypoxic Conditions: Possible Implications on the Varicose Vein Endothelium

**DOI:** 10.1155/2018/7156150

**Published:** 2018-10-18

**Authors:** Miguel A. Ortega, Beatriz Romero, Ángel Asúnsolo, Felipe Sainz, Clara Martinez-Vivero, Melchor Álvarez-Mon, Julia Buján, Natalio García-Honduvilla

**Affiliations:** ^1^Department of Medicine and Medical Specialities, Faculty of Medicine and Health Sciences, University of Alcalá, Alcalá de Henares, Madrid, Spain; ^2^Networking Biomedical Research Center on Bioengineering, Biomaterials and Nanomedicine (CIBER-BBN), Madrid, Spain; ^3^Ramón y Cajal Institute of Healthcare Research (IRYCIS), Madrid, Spain; ^4^Department of Surgery, Medical and Social Sciences, Faculty of Medicine and Health Sciences, University of Alcalá, Alcalá de Henares, Madrid, Spain; ^5^Angiology and Vascular Surgery Unit, Central University Hospital of Defense-UAH, Madrid, Spain; ^6^Immune System Diseases-Rheumatology and Oncology Service, University Hospital Príncipe de Asturias, Alcalá de Henares, Madrid, Spain; ^7^Universitary Center of Defense of Madrid (CUD-ACD), Madrid, Spain

## Abstract

Varicose veins are a disease with high incidence and prevalence. In the venous wall, the smooth muscle cells (SMCs) act in the vascular homeostasis that secretes multiple substances in response to stimuli. Any alteration of these cells can modify the function and structure of the other venous layers such as the endothelium, resulting in increases in endothelial permeability and release of substances. Therefore, knowing the cellular and molecular mechanisms of varicose veins is imperative. The aims of this study are to understand how SMCs of patients with varicose veins subjected to saphenectomy of the great saphenous vein react under hypoxic cell conditions and to determine the role of vascular endothelial growth factor (VEGF) in this process. We obtained SMCs from human saphenous vein segments from patients with varicose veins (n=10) and from organ donors (n=6) undergoing surgery. Once expanded, the cells were subjected to hypoxic conditions in specific chambers, and expansion was examined through analyzing morphology and the expression of *α*-actin. Further gene expression studies of HIF-1*α*, EGLN3, VEGF, TGF-*β*1, eNOS, and Tie-2 were performed using RT-qPCR. This study reveals the reaction of venous cells to sustained hypoxia. As significant differential gene expression was observed, we were able to determine how venous cells are sensitive to hypoxia. We hypothesize that venous insufficiency leads to cellular hypoxia with homeostatic imbalance. VEGF plays a differential role that can be related to the cellular quiescence markers in varicose veins, which are possible therapeutic targets. Our results show how SMCs are sensitive to hypoxia with a different gene expression. Therefore, we can assume that the condition of venous insufficiency leads to a situation of sustained cellular hypoxia. This situation may explain the cellular response that occurs in the venous wall as a compensatory mechanism.

## 1. Introduction

Chronic venous disease refers to morphological and functional anomalies of the venous system and includes a series of clinical manifestations of varying severity of which varicose veins (VV) are the most common [1, 2]. Within this pathology, family history, aging, hormones, obesity, and pregnancy are the most important risk factors [3–5]. The different epidemiological studies carried out worldwide have made evident that chronic venous disease is greatly variable in its incidence and prevalence. According to the Framingham study, the incidence of varicose veins per year is 2.6 % in women and 1.9 % in men [6]. In Western countries, varicose veins can affect up to 80 % of the adult population [7].

In the venous wall, the smooth muscle cells (SMCs) have an important role in the reception and control of the signaling in venous wall [8–10]. Under normal conditions in vivo, vascular cells maintain a very low replication level and lack specialized structures [11]. However, as it is able to activate and respond to numerous inflammatory, immune and thrombotic stimuli to maintain integrity, it influences the endothelium. The endothelium can perform complex functions and is vital for the maintenance of vascular wall homeostasis [12]. Endothelial cells can secrete multiple substances in response to different stimuli [13]. Any alteration of these cells can modify the function and structure of the other venous layers, resulting in the appearance of phenomena such as thrombosis, increased endothelial permeability with edema, and toxic substance release, which can lead to inflammation, ischemia, and even cell necrosis [10, 14].

Numerous authors have revealed the cellular and molecular mechanisms of chronic venous disease [10, 15–17]. Shoab et al. [18] showed that the synthesis of vascular endothelial growth factor (VEGF) is imbalanced in patients with VV, revealing its importance in the disease. VV produces distension of the venous wall and loss of normal fluid shear stress, which can lead to cellular hypoxia [14, 19].

The aims of this study are to understand how the smooth muscle cells (SMCs) of patients with varicose veins subjected to saphenectomy of the great saphenous vein react in situations of cellular hypoxia and to determine the role of VEGF in this process. We examined hypoxia, inflammation, and quiescence markers such as hypoxia-inducible factor 1 Alfa (HIF-1*α*), egl nine homolog 3 (EGLN3), VEGF, transforming growth factor beta 1 (TGF-*β*1), endothelial nitric oxide synthase (eNOS), and TEK receptor tyrosine kinase (Tie-2) to address these aims.

## 2. Patients and Methods

### 2.1. Patients

Saphenous vein segments were obtained during surgery from organ donors (controls, n=6) and subjects with venous insufficiency (varicose veins, n=10). Informed consent to participate in this study was obtained from all of the subjects. The project was approved by the Clinical Research Ethics Committee of the Central University Hospital of Defense-UAH (37/17). The specimens were first visually inspected to check for the presence of damaged areas in the vein wall. The mean of the study population was control=44,80 ± 0,86 years of age and varicose veins=47,12±1,26 years of age.

The segments of saphenous vein in the control group were obtained from organ donors, with no history of venous insufficiency or proven reflux during organ extraction surgery. Segments of saphenous vein in the second group were obtained at the time of extraction from patients with primary venous insufficiency and clinically confirmed. All varicose veins used in the study were classified as type 2 according to CEAP classification (C2).

The specimens were placed in sterile culture medium (MEM; minimal essential medium) with 1% antibiotic/antimycotic (broth from Thermo Fisher Scientific, Waltham, MA, USA) and stored at 4°C for their transfer to the laboratory, where they were divided into two fragments, one fragment was processed to obtain smooth muscle cells from explants and light microscopy (immunohistochemistry), and the other fragment was used for molecular biology studies.

### 2.2. Cells Isolation and Culture

Under sterile conditions in a Class II laminar flow cabinet (Telstar AV30/70; Telstar SA, Madrid, Spain), segments of human vein were flushed several times with MEM under sterile conditions and then longitudinally cut open. After removal of the endothelial and adventitial layers by scraping, the medial layer was cut into small explants (1 mm2). Subsequently they were subjected to digestion in a 0,1% type I collagenase solution (Worthington) in MEM (1h a 37°C) shaking in a bath. The enzyme reaction was stopped by adding the same volume of culture medium then centrifuged at 200 g for 7 min and discarded the medium. These explants were placed on the culture surface of 25 cm2 in a Roux flask (Nyclon-Intermed; Nunc A/S, Roskildo, Denmark) to which 0,5 ml Amniomax complete medium (Gibco BRL, Life Technologies Carlsbad, CA, USA) had been added to maintain the humidity of the culture surface and to improve the adherence of explants. The culture flasks were then incubated in a vertical position at 37°C in the presence of 5% CO2 in a cultured oven for 2 h. Next, 2,5 ml Amniomax medium was added per flask, and the flasks incubated horizontally under the previous conditions. Care was taken to avoid movements that might cause the explants to become unstuck. The culture medium was carefully replaced twice a week.

Once the cells had grown to confluence, SMCs were subcultured by enzyme treatment. This involved withdrawing the medium and rinsing three times in 2 ml of Hank's balanced salt solution (Gibco BRL, Life Technologies), followed by the addition of 2 ml trypsin-ethylenediaminetetraacetic acid solution at 1:250 (Gibco BRL, Life Technologies) and incubation at 37°C for 5 min. The enzyme reaction was stopped by the addition of 4 ml of culture medium. The resultant cell suspension was centrifuged at 200 g for 7 min and the cell pellet was resuspended in 9 ml of Amniomax medium. These cells in suspension were once again placed in culture at a density of 3 ml per 25 cm2 Roux flask until a confluent monolayer was obtained in an incubator with humidified 5% CO2 atmosphere at 37°C.

After that, cells were trypsinized as above and they were seeded in 12 mm diameter round glass coverslips (Nunclon Delta Surface, Thermo Fischer Scientific; Roskilde, Denmark) at the number of 30.000 cells per coverslip, and they were maintained in the humidified incubator for 48 hours prior to being subjected to hypoxic conditions.

These conditions were to establish** 4 study groups**:** Group I:** cells from healthy (CV-SMC) in normoxic conditions (NOR),** Group II:** varicose vein cells (VV-SMC) in normoxic conditions,** Group III:** CV-SMC in hypoxic conditions (HYP), and** Group IV:** VV-SMC in hypoxic conditions. The number of viable cells was determined by trypan blue exclusion and counted in a Neubauer chamber. All experiments were performed in triplicate.

### 2.3. Hypoxia Studies

In parallel experiments under normoxic conditions at 48h growth both CV- and VV-SMC cells were subjected to hypoxia in a gas-generating pouch system with indicator (GasPack EZ Gas Generating Pouches; Becton Dickinson and Company, Franklin Lakes, NJ, USA) to reduce oxygen levels to ≤1% (according to the manufacturer) during 6 hours. Hypoxic condition was confirmed with the anaerobic indicator saturated with a methylene blue solution on each sachet. This solution turns from blue to colorless in the absence of oxygen (according to the manufacturer). After the hypoxic conditions, the cells continued growing in oxygenate culture medium during more than 50 hours.

### 2.4. Alpha-Actin Immunocytochemistry

Cells from this assay were used to determine the protein expression of the a-actin. Confluent SMCs were fixed in 4% paraformaldehyde for 10 min at 4°C. Once fixed, the cells were hydrated and equilibrated twice in PBS 1X (pH 7.4). Then, cells were permeated with PBS containing 0.1% Triton X-100, 1% BSA, and 10% FBS for 45 min at room temperature. After that, primary antibody anti *α*-actin (dilution 1:400) (Sigma-Aldrich) was applied overnight at 4°C. Cells were washed three times with PBS and incubated for 1 h at room temperature with the secondary antibody anti-mouse IgG-biotin conjugate (1:300) (Sigma-Aldrich) for *α*-actin detection. Then, samples were washed three times with PBS and incubated for 90 min at room temperature with ExtrAvidin-alkaline phosphatase (1:200) (Sigma-Aldrich) for *α*-actin detection. After washing with PBS, *α*-actin was revealed with Fast Red kit (Sigma-Aldrich). Nuclei were counterstained with light hematoxylin staining. After immunostaining, the cell cultures were examined under a light microscope (Zeiss).

### 2.5. Real Time RT-PCR

RNA was extracted through guanidine-phenol-chloroform isothiocyanate procedures using Trizol (Invitrogen, Carlsbad, CA, USA) from confluent smc cultures. The RNA was recovered from the aqueous phase and precipitated by adding isopropanol and incubating overnight at -20°C. RNA integrity was checked using a 1% (w/v) agarose gel and quantified by spectrophotometry. Complementary DNA was synthesized using 200 ng of the total RNA by reverse transcription with oligo dT primers (Amersham, Fairfield, CT, USA) and the enzyme MML V-RT (Invitrogen). The following specific cDNAs were them amplified by PCR (Table 1).

The RT-PCR mixture contained 5 *µ*l of the inverse transcription product (cDNA) diluted 1:20, 10 *µ*l of iQ SYBR Green Supermix (Bio-Rad Laboratories) and 1 *µ*l (6 *µ*M of each primer in a final reaction volume of 20 *µ*l. RT-PCR was performed on a StepOne PlusTM System (Applied Biosystems-Life Technologies), using the relative standard curve method [20]. Samples were subjected to an initial stage of 10 min at 95°C. The conditions for cDNA amplification were 40 cycles of 95°C for 15s, 59°C (HIF-1*α*) or 60°C (EGLN3, TGF-*β*1, VEGF, eNOS, Tie-2 and GAPDH) for 30 s and 72°C for 1 min and a final stage of 15 s at 95°C, 1 min at 60°C, 15 s at 95°C and 15 s at 60°C. Fluorescence was determined at the end of each cycle. The data obtained from each gene are interpolated in a standard curve made by serial dilutions of a mixture of the study samples which is included in each plate. Gene expression was normalized against the expression recorded for the reference GAPDH gene. All tests were performed in triplicate. Results were expressed in Relative Quantity mRNA (RQ).

### 2.6. Statistical Analysis

For the statistical analysis, the GraphPad Prism® 5.1 program was used, applying the Mann–Whitney U test. The data are expressed as the mean ± deviation from the mean. The significance is set at p <0.05 (*∗*), p <0.005 (*∗∗*), and p <0.001 (*∗∗∗*).

## 3. Results

### 3.1. The Effects of Hypoxia on Smooth Muscle Cells from Healthy (CV-SMCs) and Varicose (VV-SMCs) Veins

**Figure 1:**
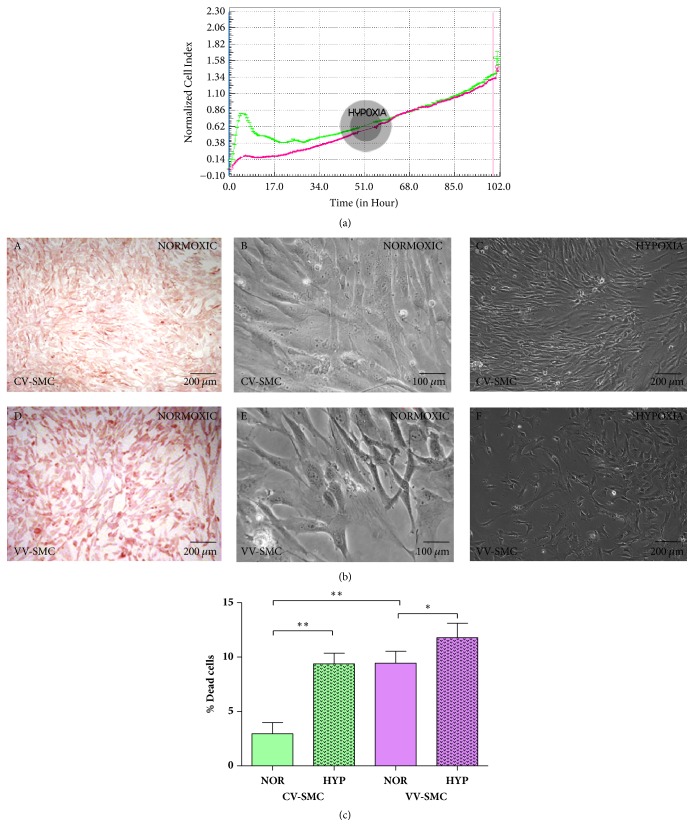
(a) Normalized cell index of cultures with control smooth muscle cells (CV-SMCs, green line) and varicose veins smooth muscle cells (VV-SMCs, purple line) veins under hypoxic conditions. (b) Images of CV-SMCs and VV-SMCs in which the differential expression of *α*-actin (A AND D) can be observed. Different morphology and proliferation were observed for the CV-SMCs and VV-SMCs under normoxic (B AND E) and hypoxic conditions (C AND F) via inverted microscopy. (c) Quantification of the percentage of dead cells in the CV-SMCs and VV-SMCs under normoxic (NOR) and hypoxic conditions (HYP). p<0.05 (*∗*); p<0.005 (*∗∗*).

First, we investigated the behavior of smooth muscle cells from healthy and varicose veins during their expansion under* in vitro *conditions. We observed that, in oxygenated culture medium, CV-SMCs showed better adhesion and proliferation than those of VV-SMCs during the first eight hours (Figures 1(a) and 1(b)). The CV-SMCs also had higher protein expression levels of *α*-actin (Figure 1(b)). Subsequently, the CV-SMCs and VV-SMCs were subjected to experimental hypoxic conditions. Under these conditions, both cell populations were observed to have similar behavior (Figure 1(b)).

When studying cell behavior, differences were observed in the number of dead cells in both groups (Figure 1(c)). Under conditions of hypoxia (HYP), the CV-SMCs showed a significant increase in the percentage of dead cells with respect to the normoxic condition (NOR) (2.96±1.02 % NOR versus 9.38±0.97 % HYP, *∗∗*p<0.005). A significant increase was also observed in the percentage of dead cells under hypoxic conditions for the VV-SMCs (9.44±1.08 % NOR versus 11.78±1.33 % HYP, *∗*p<0.05). When comparing both of the study groups under normoxic conditions, the VV-SMCs showed a significant increase in the percentage of dead cells with respect to the CV-SMCs (*∗∗*p<0.005).

### 3.2. Expression of Hypoxia Markers

**Figure 2:**
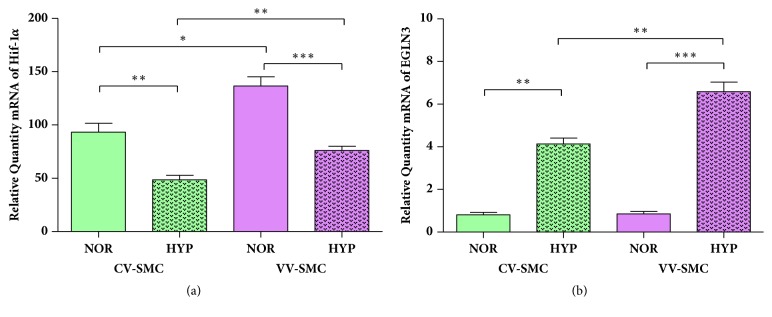
mRNA levels of HIF-1*α* (a) and EGLN3 (b) in smooth muscle cells from healthy (CV-SMCs) and varicose veins (VV-SMCs) patients under normoxic (NOR) and hypoxic (HYP) conditions. The results were normalized to that of the GAPDH gene and are provided in arbitrary units. The data are expressed as the mean ± standard deviation from the mean. The significance is set at p<0.05 (*∗*), p<0.005 (*∗∗*), or p<0.001 (*∗∗∗*).

The expression of HIF-1*α* and EGLN3 was examined, and our results showed significant differences in the expression of these genes in the study groups under conditions of experimental normoxia and hypoxia (Figure 2).

For** HIF-1α**, the CV-SMCs under normoxic conditions were observed to have levels significantly higher than those under hypoxic conditions (93.17±8.42 RQ NOR versus 48.61±4.09 RQ HYP, *∗∗*p<0.005). The VV-SMCs under normoxic conditions showed significantly higher levels than in the hypoxia group (136.59±8.72 RQ NOR versus 76.07±4.04 RQ HYP, *∗∗∗*p<0.001). When comparing the CV-SMCs versus VV-SMCs groups, we observed significantly higher expression levels under normoxic condition (*∗*p<0.05) and experimental hypoxic conditions in VV-SMCs (*∗∗*p<0.005) (Figure 2(a)).

When studying the gene expression of** EGLN3, **a statistically significant elevation was found in the CV-SMCs under conditions of experimental hypoxia (0.81±0.11 RQ NOR versus 4.14±0.27 RQ HYP, *∗∗*p<0.005). A similar trend was observed for the VV-SMCs during experimental hypoxia, though the levels were significantly higher than those in the CV-SMCs (0.86±0.12 RQ NOR versus 6.59±0.44 RQ HYP, *∗∗∗*p<0.001). When comparing the study groups, statistically significant differences were established in the expression levels of EGLN3 under conditions of experimental hypoxia between the CV-SMCs and VV-SMCs (*∗∗*p<0.005) (Figure 2(b)).

### 3.3. Expression of Angiogenesis and Proliferation Markers

**Figure 3:**
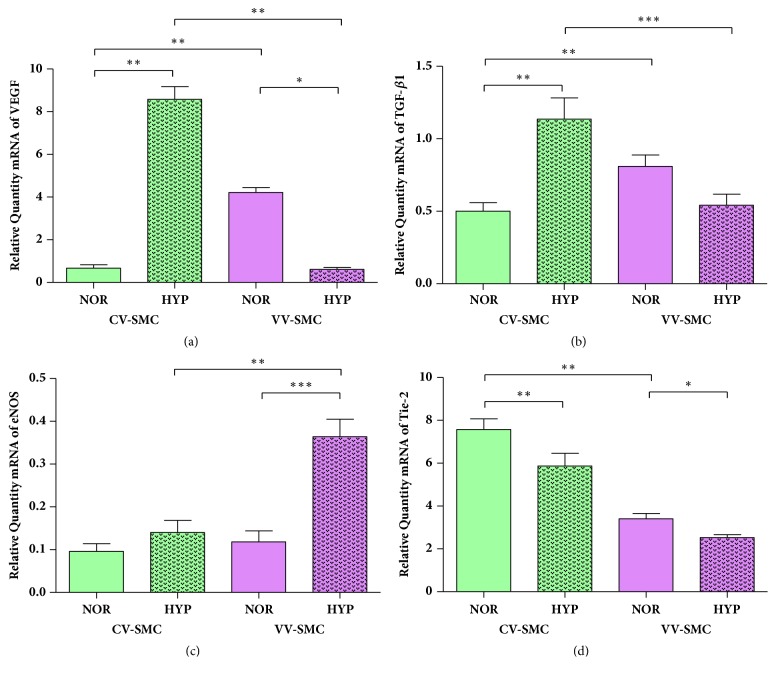
mRNA levels of VEGF (a), TGF-*β*1 (b), eNOS (c), and Tie-2 (d) in smooth muscle cells from healthy (CV-SMCs) and varicose veins (VV-SMCs) patients under normoxic (NOR) and hypoxic (HYP) conditions. The results were normalized to that of the GAPDH gene and are provided in arbitrary units. The data are expressed as the mean± standard deviation from the mean. The significance is set at p<0.05 (*∗*), p<0.005 (*∗∗*), or p<0.001 (*∗∗∗*).

To analyze angiogenesis and proliferation, the gene expression of VEGF, TGF-*β*1, eNOS, and Tie-2 was studied using RT-qPCR under conditions of experimental normoxia and hypoxia* in vitro*. Our results showed that the expression of these markers is significantly different in the two study groups (Figure 3).

The expression of** VEGF** showed a significant increase in the CV-SMCs under conditions of experimental hypoxia (0.67±0.16 RQ NOR versus 8.58±0.59 RQ HYP, *∗∗*p<0.005). In addition, in the VV-SMCs, VEGF showed a significant decrease under experimental hypoxia (4.21±0.23 RQ NOR versus 0.61±0.09 RQ HYP, *∗*p<0.05). Under conditions of normoxia, the VV-SMCs were observed to have significantly higher expression levels of VEGF compared to the CV-SMCs (*∗∗*p<0.005). When comparing both study groups, the VV-SMCs showed a significant decrease in VEGF expression compared to that of the CV-SMCs under conditions of experimental hypoxia (*∗∗*p<0.005) (Figure 3(a)).

When** TGF-β1** was studied, the CV-SMCs were observed to have a significant increase in expression under conditions of experimental hypoxia (0.50±0.06 RQ NOR versus 1.14±0.15 RQ HYP, *∗∗*p<0.005). For the VV-SMCs, no statistically significant differences were observed in the expression levels of TGF-*β*1 under conditions of experimental normoxia and hypoxia (0.81±0.08 RQ NOR versus 0.54±0.07 RQ HYP). When comparing both study groups, the VV-SMCs had significantly higher TGF-*β*1 expression levels than those of the CV-SMCs under normoxic conditions (*∗∗*p<0.005). Under experimental hypoxic conditions, these levels were significantly lower in the VV-SMCs (*∗∗∗*p<0.001) (Figure 3(b)).

The expression levels of** eNOS** were not significantly different in the CV-SMCs (0.09±0.02 RQ NOR versus 0.14±0.03 RQ HYP). However, when studying the VV-SMCs, a significant increase in eNOS expression was observed under conditions of experimental hypoxia (0.11±0.02 RQ NOR versus 0.36±0.04 RQ HYP, *∗∗∗*p<0.001). Comparing both study groups, the expression levels of eNOS in the VV-SMCs were statistically significantly higher than those of the CV-SMCs under conditions of experimental hypoxia (*∗∗*p<0.005) (Figure 3(c)).

Finally,** Tie-2** was studied, and a significant decrease in its expression levels in the CV-SMCs was observed under conditions of experimental hypoxia (7.57±0.49 RQ NOR versus 5.87±0.59 RQ HYP, *∗∗*p<0.005). In the VV-SMCs, a significant decrease in the Tie-2 expression levels was also observed with experimental hypoxia (3.40±0.25 RQ NOR versus 2.53±0.14 RQ HYP, *∗*p<0.05). Furthermore, in conditions of normoxia, the VV-SMCs were found to have to significantly less Tie-2 than the CV-SMCs (*∗∗*p<0.005) (Figure 3(d)).

## 4. Discussion

Of the various noxae that can affect the venous wall and its function, as shown in previous studies, homeostatic balance and the ability to react to different conditions contribute to venous failure [15, 16, 21, 22]. Venous insufficiency (varicose veins) produces venous wall dilatation, which leads to alterations in the structure of the compensatory wall, first in the form of areas of hypertrophy that, subsequent to failure, will become fibrosclerotic at the end of the process [13]. These induced alterations, encouraged and maintained by ischemic phenomena, lead to cellular activation, in turn causing repercussions at the functional level of the venous wall.

The ischemic process plays an important role in the process of venous wall insufficiency [23, 24]. Cellular behavior allows us to infer that microvascular dysfunction is the main alteration that should be considered [21]. The important role of VEGF in vascular pathology has been highlighted by numerous authors, with studies mentioning its role in angiogenesis and cell signaling [25, 26]. Bjarath et al. [27] noted that VEGF can cause remodeling of the venous wall in patients with varicose veins and that it could be associated with a genetic component. VEGF has been shown to play a role in the cellular response in different tissues in patients with venous insufficiency, conditioning the activity and response of affected tissue, such as the skin, in reepithelization processes in venous ulcers [25, 28]. Our results show that, in the controls, the expression of VEGF is significantly higher under hypoxic conditions compared to normoxic conditions. However, importantly, the levels of VEGF are significantly higher in SMCs from patients with varicose veins during normoxia, and they decrease under conditions of long-term hypoxia. These findings suggest that cells from patients with varicose veins suffering from hypoxia could be in a state of quiescence and could have significantly higher markers such as Tie-2. Some authors have reported that Tie-2 plays a role in the process of quiescence in vascular pathology, whereas in homeostasis, Tie-2 is a potential angiogenic factor [29]. VEGF can affect the permeability of the venous wall and directly influence eNOS levels, which could explain the increased expression of this molecule under conditions of hypoxia and venous insufficiency [30].

Inflammation is another process factor that has been indicated to be essential for the progression of varicose veins [31]. Jin et al. [32] showed a strong relationship between the actions of VEGF and TGF-*β*1 in the pathophysiological processes that give rise to tissue damage. Moreover, VEGF and TGF-*β*1 have been shown to have different levels of gene expression depending on the conditions to which the cells are exposed [33]. Our results showed similar relative expression profiles for VEGF and TGF-*β*1; however, in absolute terms, the expression levels of VEGF were higher than all of the other studied markers.

Our results show that HIF-1*α* is differentially expressed in the SMCs from the study patients and that its expression is inversely related to the expression of EGLN3. Numerous authors have postulated that this pathway for hypoxia is deregulated in chronic venous disease, which causes the deregulation of the expression of these angiogenic factors [34–38]. In relation to this fact, EGLN3 has been shown to play an important role as an adsorber to hypoxic tissue and is also important for homeostasis [39]. Lee et al. [34, 35] described a significant imbalance of HIF-1*α* in the muscle layers of diseased vessels.

Knowing the cellular behavior of venous wall cells under conditions of normoxia and hypoxia is fundamental for understanding the venous wall as well as for identifying possible therapeutic targets that should be studied. The present study, despite having a preliminary character, should be considered that the cells are homogenized in the same culture conditions. The detection of significant differences between the studied populations acquires a great relevance for subsequent* in vivo *studies. Therefore, we can assume that the condition of venous insufficiency leads to a situation of sustained cellular hypoxia. This situation may explain the cellular response that occurs in the venous wall as a compensatory mechanism. In this study, we demonstrated two important facts. First, the muscle cells of people with varicose veins show levels of the markers studied similar to normal cells subjected to hypoxia (Hif-1*α*, VEGF, TGF-*β*1, and eNOS). That is, these cells have stable genetic or epigenetic changes, which are maintained after their stabilization in the culture medium. Secondly, we show that if this hypoxia continues in the long term, these cells no longer have the capacity to react and there is a failure of the factors that try to compensate the hypoxia (EGLN3). This fact would allow us to establish a correlation between the presence of certain markers in the patient's serum and the ability to react to sustained hypoxia. At this point, the new microRNA technology could help establish such a correlation.

## Figures and Tables

**Table 1:** RT-qPCR primer sequences and binding temperatures (Temp).

**GENE**	**SEQUENCE Fwd (5**′**→3**′**)**	**SEQUENCE Rev (5**′**→3**′**)**	**Temp**
**HIF-1** **α**	ACGTGTTATCTGTCGCTTTGAG	ATCGTCTGGCTGCTGTAATAATG	59°C

**EGLN3**	GATGCTGAAGAAAGGGC	CTGGCAAAGAGAGTATCTG	60°C

**TGF-** **β** **1**	GCGTGCTAATGGTGGAAAC	CGGAGCTCTTGATGTGTTGAAGA	60°C

**VEGF **	ATGACGAGGGCCTGGAGTGTG	CCTATGTGCTGGCCTTGGTGAG	60°C

**eNOS **	AAG AGG AAG GAG TCC AGT AAC ACA GA	ACG AGC AAA GGC GCA GAA	60°C

**Tie-2 **	TGCCCAGATATTGGTGTCCT	CTCATAAAGCGTGGTATTCACGTA	60°C

**GAPDH **	ATGACGAGGGCCTGGAGTGTG	CCTATGTGCTGGCCTTGGTGAG	60°C

## Data Availability

The data used to support the findings of this study are available from the corresponding author upon request.
